# Physical activity intervention (Movi-Kids) on improving academic achievement and adiposity in preschoolers with or without attention deficit hyperactivity disorder: study protocol for a randomized controlled trial

**DOI:** 10.1186/s13063-015-0992-7

**Published:** 2015-10-12

**Authors:** Mairena Sánchez-López, María Jesús Pardo-Guijarro, David Gutiérrez-Díaz del Campo, Pedro Silva, Maria Martínez-Andrés, Roberto Gulías-González, Ana Díez-Fernández, Pablo Franquelo-Morales, Vicente Martínez-Vizcaíno, On behalf of the Movi-Kids group

**Affiliations:** Health and Social Research Centre, Universidad de Castilla-La Mancha, Edificio Melchor Cano, C/ Teresa Jornet s/n, 16071 Cuenca, Spain; Faculty of Education, Universidad de Castilla-La Mancha, Ciudad Real, Spain; Faculty of Education, Universidad de Castilla-La Mancha, Cuenca, Spain; Faculty of Sport, Research Centre in Physical Activity, Health and Leisure, University of Porto, Porto, Portugal; Faculty of Occupational Therapy, Speech Therapy and Nursing, University of Castilla-La Mancha, Talavera de la Reina, Spain; Emergency Department, Hospital Virgen de la Luz, Cuenca, Spain; Facultad de Ciencias de la Salud, Universidad Autónoma de Chile, Talca, Chile

**Keywords:** academic achievement, attention deficit hyperactivity disorder, children, health-related quality of life, motor skill, Movi-Kids, obesity, physical activity, quality of sleep

## Abstract

**Background:**

The prevention of obesity and improvement of academic achievement in children are concerns of industrialized societies. Obesity has been associated with psychological disorders, including attention deficit hyperactivity disorder, whose prevalence has been estimated at 6.8 % in Spanish children and adolescents. It is known that physical activity is positively related to academic achievement and negatively related to the risk of obesity in children. However, studies to test the effectiveness of physical activity interventions in improving academic achievement in preschool children are scarce and have some weaknesses that threaten their validity. Moreover, very few studies have examined their effectiveness in improving symptoms of attention deficit hyperactivity disorder. This paper outlines a two-year multidimensional preschool intervention (Movi-Kids) aimed at preventing obesity and improving academic achievement in children with or without attention deficit hyperactivity disorder.

**Methods/Design:**

Twenty-one schools from Ciudad Real and Cuenca, Spain, were randomized to intervention and control groups. In the first academic year, children in the third grade of preschool and the first grade of primary school in the intervention group received the Movi-Kids intervention. In the second academic year, schools were crossed over to the other group. The intervention included children, parents and teachers, and the school environment, and consisted of: (i) three hour-long sessions of recreational non-competitive physical activity after school, weekly, (ii) educational materials for parents and teachers addressing sedentary lifestyle risks and (iii) playground modifications to promote physical activity during breaks. Primary outcome measures of this study were academic achievement (intelligence, cognition, memory, attention and perception), assessed by the Battery of General and Differential Aptitudes, and adiposity measures (body mass index, waist circumference, triceps skinfold thickness and body fat percentage). Secondary outcome measures were: attention deficit hyperactivity disorder risk, motor skills, health-related quality of life and sleep quality. These variables will all be measured in both groups at baseline and at the end of the first and second academic years.

**Discussion:**

It seems reasonable that an intervention to promote physical activity based on playground games will be useful for simultaneously improving academic achievement and controlling obesity.

**Trial registration:**

ClinicalTrials.gov NCT01971827.

## Background

The analysis of the relationship between physical activity and academic achievement is an emerging concern, owing to the alarming decrease in physical activity levels in schoolchildren at young ages and social pressure to achieve academically. Physical activity and fitness have been positively related to academic achievement in schoolchildren [1, 2]. This association has been explained by the structural changes that physical exercise generates in the brain, such as neurogenesis, angiogenesis, increased hippocampal volume and connectivity [3, 4].

Many cross-sectional studies support a positive relationship between physical activity, fitness, cognitive development and academic achievement in children and adolescents [1, 5]. However, a recent systematic review [6] of randomized controlled trials warns of the scarcity of studies that assess the effectiveness of interventions promoting physical activity in order to improve academic achievement or cognitive skills in children and adolescents. Moreover, this review [6] concludes that more rigorous trials using adequate sample sizes, standardized interventions, valid and reliable tools of measurement, and long-term follow-up for sustained cognitive and psychosocial outcomes are needed.

The figures relating to childhood obesity in Spain and other Western countries represent a major public health concern [7, 8]. The current prevalence of overweight and obesity in children aged 6 to 8 years from Castilla-La Mancha is 36.8 % [9], similar to other areas in Spain [10]. Obese children are at increased risk of becoming obese adults [11, 12], and the risk of adult obesity is much greater, as earlier adiposity rebound occurs [13, 14], a crucial period in the development of obesity. Excess weight in childhood is associated with different cardiovascular risk factors [15] and other health problems, such as obstructive sleep apnoea [16], psychological disorders [17], low academic achievement [16] and poor quality of life [18]. However, the interaction between genes, environment and lifestyle factors has been considered critical in the development of childhood obesity [19, 20]; among lifestyle factors, physical activity plays a central role in the prevention and treatment of this problem [21, 22].

The latest Cochrane review [23] highlights that there is consistent evidence regarding the effectiveness of interventions for preventing obesity in children aged 6 to 12 years. However, studies that address the effectiveness of such interventions in children younger than 6 years are scarce [23]. Only one study analyzed a healthy lifestyle promotional intervention (Ballabeina) in predominantly migrant preschool children, with successful effectiveness in reducing adiposity and improving fitness [24].

According to the Diagnostic and Statistical Manual of Mental Disorders, fifth edition (DSM-5) [25], attention deficit hyperactivity disorder (ADHD) is characterized by a persistent and impairing pattern of inattention, hyperactivity or impulsivity. A consistent body of evidence confirms that ADHD is often associated with other psychiatric conditions, such as specific learning disorders, mood and anxiety disorders, and sleep disturbances [26]. The aetiology of ADHD is complex and multidimensional, and combines both genetic and environmental factors. However, early diagnosis and appropriate treatment can positively influence its evolution [26–28]. In terms of prevalence, ADHD is one of the most common neurodevelopmental disorders in early childhood, affecting from 4.9 to 8.8 % of children and adolescents in Spain [29].

Interest has grown in the comorbidity between ADHD and other psychiatric and medical conditions. Clinical and epidemiological studies have consistently associated ADHD with overweight and obesity [30, 31]. Three mechanisms have been proposed to explain this relationship: (1) it has been suggested that two obesity-related factors, binge eating and excessive daytime sleepiness, might occur in children with ADHD, (2) obesity and ADHD share genetic and neurobiological abnormalities, such as dysfunctions in brain reward pathways, and (3) impulsivity and inattention are associated with irregular and dysregulated eating patterns, which contribute to weight gain [31].

Conversely, several researchers have described the relationship among physical exercise and stress, anxiety, depression, behavioural problems, impulse control, improvement of interpersonal relationships, academic achievement and working memory in ADHD children [32–34]. These benefits of physical activity in children with ADHD might be mediated by changes in levels of stress hormones and serotonin, although evidence in this regard is inconclusive, since, as a recent meta-analysis [35] underlines, very few studies have examined the effect of physical activity interventions on symptoms such as inattention, hyperactivity or impulsivity, anxiety and cognitive functions in children with ADHD, and those studies have all been conducted in children older than 7 years.

Thus, this paper reports on the rationale and methods of a trial aimed at assessing the effectiveness of a multidimensional physical activity intervention (Movi-Kids) for improving academic achievement and adiposity in school children, with or without ADHD, aged 4 to 7 years from Castilla-La Mancha, Spain. The secondary objectives will be to estimate the prevalence of ADHD in this age group of schoolchildren, and to assess the effectiveness of this programme for improving children’s levels of physical activity (during break time), motor skills, health-related quality of life and sleep quality.

This project is coordinated with another project that shares the same population-based sample, which is aimed at assessing the effectiveness of a physical activity intervention for preventing obesity and reducing cardiovascular risk during the adiposity rebound period in children from Castilla-La Mancha, Spain (ClinicalTrials.gov: NCT01971840) [36].

## Methods/Design

### Study design and participants

**Fig. 1:**
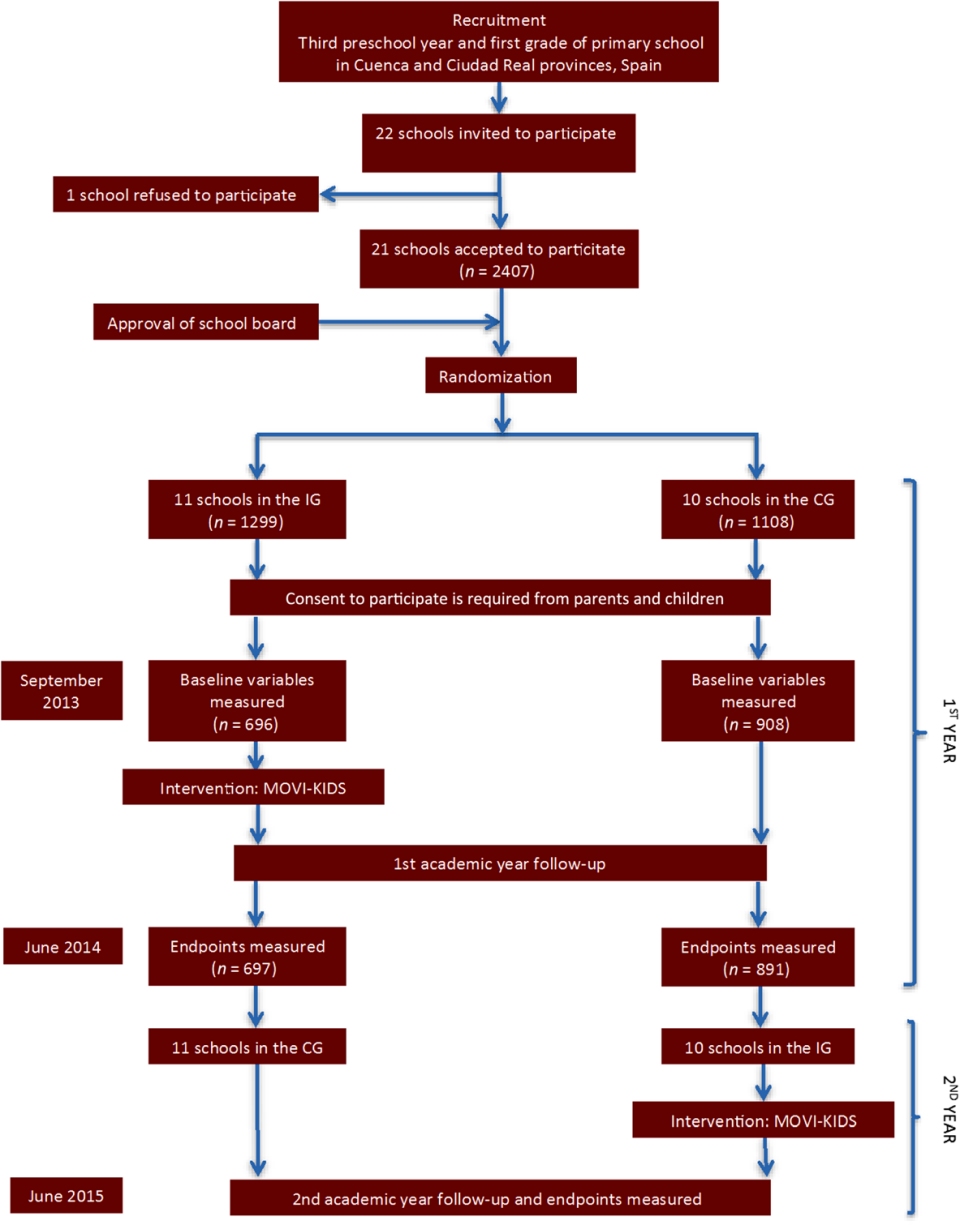
Flow chart of trial participants. CG, control group; IG, intervention group

A crossover randomized cluster trial was carried out, involving 21 schools located in as many municipalities in the provinces of Cuenca and Ciudad Real, in the Castilla-La Mancha region of Spain. All of them were state-funded schools except for two, which were private. In municipalities with more than one school, only one was selected for the study, to avoid contamination of the intervention. At first, 22 schools were invited, and only one of them refused to participate in the study, arguing that the study could mean excessive administrative overheads for the teachers. After the approval of school councils, the schools were randomly allocated using the statistical package StatsDirect to either the intervention or the control group; finally, the whole sample of schools was divided into three subgroups of randomization as follows: (i) nine public schools from Cuenca, (ii) ten public schools from Ciudad Real, and (iii) two private schools, one in the capital of each province (Fig. 1). All children belonging to the third grade of preschool and first grade of primary school (aged 4 to 7 years) were invited to participate.

#### Inclusion criteria

All participating schools had to have at least two full classrooms for both the third year of preschool and the first grade of primary school. To participate in the intervention and in the measurements at the beginning and end of each academic year, the approval of boards of governors was necessary. All children’s parents or legal representatives signed an informed consent to participate, and were invited to collaborate by filling in questionnaires with regard to family leisure habits, sleeping, eating and getting around town. They completed the questionnaire at home and returned it to the teachers one week later.

#### Exclusion criteria

Children who had any of the following conditions were excluded: (a) severe Spanish language learning difficulties, (b) serious physical or mental disorders identified by parents or teachers that would impede participation in the programme’s activities, or (c) those with diagnoses of chronic disorders, such as heart disease, diabetes or asthma, which in the opinion of their paediatricians would prevent their participation in the programme’s activities.

Schools with only one full third-grade class of preschool or one first-grade class of primary school were excluded.

#### Ethical and legal aspects

The project had the explicit support of the Department of Education and Science of the Junta de Comunidades of Castilla-La Mancha, Spain, who sent a letter to each school that included information about the study. Next, investigators visited each school to explain the aims and methods of the study and to obtain the consent of the head teacher and the school board. This was followed by classroom-by-classroom meetings, in which pupils were asked to collaborate. The Movi-Kids study was also presented to teachers of physical education. Through the teachers, a letter was sent to parents inviting them to a group meeting at the school. In this meeting, the objectives, measurements and procedures of the study were explained, and responses provided to any questions and objections that were raised. Moreover, in this session, informed consent was obtained from all parents for the participation of their children in the study. This document was provided to teachers at the beginning of each academic year.

After each evaluation process, letters with the results of adiposity measures of their children were sent to parents. In those children in which any unhealthy value was detected (e.g., overweight or obesity), the medical research team personally telephoned with parents to give them appropriate recommendations. It was not possible to inform parents immediately about other study variables (e.g., academic achievement or risk of ADHD) because these questionnaires require a more complex analysis.

The study protocol was approved by the clinical research ethics committee of the Virgen de la Luz Hospital in Cuenca and General Universitario Hospital in Ciudad Real. An insurance policy was also signed to cover any potential risks associated with the intervention.

### Study variables and measurements

Baseline and post-intervention outcome variables were measured in both the control and intervention groups three times, at the beginning and end of the first academic year (in September 2013 and June 2014), and at the end of the second academic year (in June 2015). Table 1 provides an overview of all variables measured. Anthropometry (weight, height, body mass index, waist circumference, triceps skinfold thickness, percentage of body fat and fat-free mass) was performed in standardized conditions, as extensively described in the MOVI-2 protocol [37].

**Table 1:** Study variables

Type of variable	Specific variables
Primary endpoint measures	Anthropometry: weight, height, body mass index, waist circumference, triceps skinfold thickness, percentage of body fat and fat-free mass by bioelectrical impedance analysis
	Academic achievement
Secondary endpoint measures	Attention deficit hyperactivity disorder risk: parents’ and teachers’ report using the Attention Deficit Hyperactivity Disorder Rating Scale-IV
	Motor skills: fine motor skills, throws and catches, and balance (static and dynamic), assessed by the Movement Assessment Battery for Children
	Health-related quality of life: children’s (by interview) and parents’ (self-administered) report, using the Kiddy-Kindl Questionnaire
	Sleep quality: parents’ report using the Children’s Sleep Habits Questionnaire, accelerometers, children’s sleep diary
Other endpoint measures	Physical activity: parents’ report by the Netherlands Physical Activity Questionnaire, accelerometers
Potential confounding factors	Age
	Sex
	Birthweight
	Breastfeeding: breastfeeding, formula feeding or both (mixed feeding)
	Food consumption: parents’ report, using the Children’s Eating Habits Questionnaire
	Family socio-economic status: education, occupation and socio-economic status index
	Area (urban or rural)
	Origin (native or foreign)

To minimize interobserver variability, the measurements were carried out in the school by trained investigators.

#### Primary outcome measures

##### Anthropometry and body composition

Weight was measured twice (Seca® 861 scales) with the child barefoot and in light clothing. Height was also measured twice, using a wall stadiometer (Seca® 222), with the child barefoot and upright and with the sagittal midline touching the back board. Body mass index was calculated as weight in kilograms divided by the square of the height in metres. Waist circumference was measured three times at the midpoint between the last rib and the iliac crest at the end of a normal expiration and using a flexible tape. Triceps skinfold thickness was measured three times at the triceps using a Holtain Ltd. caliper (0.2 mm accuracy and consistent pressure between valves of 10 g/mm^2^). The percentage of body fat and the fat-free mass were estimated with a four-electrode Tanita® Segmental-418 bioimpedance analysis system (Tanita Corp. Tokyo, Japan). Two readings were obtained in the morning, under controlled temperature and humidity conditions, with the child shoeless and fasting, and after urination and a 15-minute rest. The mean of all the anthropometric measurements was calculated and considered for the analysis.

##### Academic achievement

Basic psychological processes involved in learning (intelligence, cognition, memory, attention and perception) were assessed using the Battery of General and Differential Aptitudes scales for children aged 3–6 years [38] and 6–8 years [39]. Both scales include: (a) global academic predictors (e.g., general intelligence), (b) non-verbal tests (e.g., reasoning and logical puzzle figures), (c) verbal tests (e.g., numerical quantitative concepts) and (d) additional tests (e.g., auditory perception).

#### Secondary outcome measures

##### Attention deficit hyperactivity disorder risk

We used the Attention Deficit Hyperactivity Disorder Rating Scale-IV, which has been translated into a Spanish version and validated [40]. It is a screening and evaluation scale of ADHD that includes two subscales, for inattention and hyperactivity, and a total score. Each item represents each of the ADHD symptoms, according to the criteria of the Diagnostic and Statistical Manual of Mental Disorders [41]. We used the parents’ and teachers’ version, which fits the criterion of ‘the presence of symptoms in at least two environments’.

##### Motor skills

These were assessed by the Movement Assessment Battery for Children [42–44]. This battery has been validated to identify and describe deficiencies in motor performance in children and adolescents from 3 to 16 years old. It consists of eight tests for each age group (3–6, 7–10 and 11–16 years) measuring three dimensions: fine motor skills, throws and catches, and balance (static and dynamic). A higher score indicates better motor performance. It enables classification of: children with motor problems, children with motor risk and children with normal motor development.

##### Health-related quality of life

This is assessed using the Kiddy-Kindl Questionnaire, which has been validated in a Spanish version for children aged 4–7 years and their parents [45]. The Kiddy-Kindl is a generic health-related quality-of-life instrument for children and adolescents, and was developed in Germany for use both in clinical practice and with healthy children. This questionnaire contains 12 questions (with three response options ranging from 1 to 3, where 1 = never, 2 = sometimes and 3 = many times) in six dimensions: physical, emotional, self-esteem, family, friends and school. The children’s version was administered by interview, the parents were offered a self-administered version.

##### Sleep quality

This is assessed using the Spanish version of the Children’s Sleep Habits Questionnaire [46], completed by parents. This instrument focuses on common sleep disorders for children aged 4 to 10 years. The questionnaire allows parents to indicate, for each item, whether they consider sleep habits are a problem for their child. It also includes four questions about the time children go to bed, wake up and get up, and the total number of hours of sleep. Finally, latency, amount, duration of sleep and number of awakenings were also measured by accelerometers (ActiSleep®) in a subsample of 60 schoolchildren. Over a week, children completed a sleep diary.

#### Other study endpoint measures

##### Physical activity

This was evaluated using the Spanish version of the Netherlands Physical Activity Questionnaire [47] for parents, in which they provide information about their children’s daily activity preferences during the six previous months. In addition, children from six schools in the intervention group and another four schools in the control group wore GT3X accelerometers for seven days consecutively (including nights) to measure their physical activity in the first academic year. The accelerometers were programmed in epochs of one second and data will be analyzed using ActiLife software, version 6.1.

#### Confounding variables

Age, sex, birthweight, breastfeeding, food consumption, socio-economic status, living area (urban or rural) and origin (native or foreign) were considered potential confounding variables.

##### Breastfeeding

Mothers were first asked what type of feeding had been chosen during their child’s infancy: breastfeeding, formula feeding or both (mixed feeding). They were also asked up to what age the child was exclusively breastfed and when breastfeeding was completely stopped. In exclusive breastfeeding, the infant received only breast milk without any supplementary feeding.

##### Food consumption

This was estimated using the Spanish version of the Children’s Eating Habits Questionnaire [48], validated for children from 2 to 9 years old. This version was completed by parents.

##### Family socio-economic status

Data regarding family socio-economic status were gathered by using self-reported occupation and education questions completed by either the father or mother. Paternal and maternal education were classified separately as primary education (functionally illiterate, without any studies or those who had not completed primary education), middle education (primary education and high school or secondary education or ‘Bachillerato’) and university education (university degree or Ph.D.). Parental occupation was classified into five categories as follows: (1) supervisor, manager or freelance with ten employees or more, (2) supervisor, manager or freelance with fewer than ten employees, (3) freelance with no staff, (4) unqualified staff and unskilled workers, and (5) household chores, unemployed or others. An index of socio-economic status was calculated using the items regarding parents’ education and occupation. This index distinguishes, according to the scale proposed by the Spanish Society of Epidemiology, five categories of family socio-economic status: lower, upper lower, lower middle, upper middle and upper [49].

### Study intervention

The design of Movi-Kids is based on the social ecological model [50], a theoretical model of behaviour change in which behaviour is understood as the interaction between the physical and social environment. The Movi-Kids programme is a multidimensional intervention aimed at influencing individuals (children, families and teachers) and the environment (including some changes in the physical structure of the playground).

The intervention was applied in the intervention group over two full academic years, and was implemented at three levels:

1. Children participated in an optional extracurricular, play-based, non-competitive physical activity programme (Movi-Kids), adapted to their levels of motor competence (from 4 to 7 years old). The aim of this programme was to increase the weekly physical activity time through three 60 min sessions per week using school facilities. In addition, the Movi-Kids programme included basic sports games, playground games, dance and other activities focused on developing motor skills. At the end of the first year, approximately 90 sessions had been carried out in each school.
2. Parents and teachers included in the intervention group were involved in activities to promote active lifestyles in their children. These activities included: (a) use of reinforcement tools that had demonstrated their utility in improving parents’ and teachers’ compliance and involvement in the programme (e.g., a refrigerator magnet with recommendations for physical activity for children), (b) answering a satisfaction-with-the-programme questionnaire, and (c) access to a blog (http://movi3kids.blogspot.com.es/) where parents could observe their children’s progress, read news regarding reinforcing healthy lifestyles, and ask questions of or make complaints to the research team.
3. Finally, environmental interventions were conducted in the playground. Fixed (a balance circuit and panels with incentives to be physically active during break time) and mobile equipment (tyres of different colours and sizes) were put in the playgrounds to encourage children to be more active during playtime.

The standard physical education curriculum (1 hour per week of psychomotor activities to third-grade preschoolers and 2 hours per week of physical education to first-grade primary schoolers with physical activity levels at low-to-moderate intensity) was applied in both the control and intervention schools because this is compulsory in Spain.

Since this was a cluster-crossover trial, clusters (schools) were randomized to intervention and control groups, with outcomes measured after the first period (first academic year), and then, after a washout period (the summer holidays), clusters crossed over from control to intervention groups, and vice versa.

#### Organization and functioning of the Movi-Kids programme

The Movi-Kids programme has been coordinated by two physical activity sciences graduates, and implemented by monitors with technical qualifications in physical activity and sports, physical education teachers or physical activity sciences graduates. The monitors were trained over two days, to standardize the actions of the activities programme.

#### Evaluation and follow-up

A telephone number, an email address and the blog (http://movi3kids.blogspot.com.es/) were available for parents and teachers over the two years. In addition, two meetings were conducted with monitors, at baseline and three months later. In each session, the monitors signed the list of participants to confirm who had attended the programme, and each month they gave a list of absentees to the programme’s coordinators by phone and email. When a child missed a programme’s sessions too often, the team called the family to find out the reasons. Finally, a quarterly visit to the centres was made to assess programme performance and conduct satisfaction surveys among the children. To encourage adherence to the Movi-Kids programme, children attending a minimum of 80 % of the trimester sessions were given positively reinforcement and received small gifts depicting the logo with the mascot of the programme.

#### Intervention with parents and teachers

Before the beginning of the programme, researchers, teachers and parents were asked to decide on the best way to implement modifications in the playground, and to discuss strategies to encourage family support of Movi-Kids. During the programme, several actions were taken to engage teachers and parents of the intervention group in promoting healthy lifestyles in children, as described.

### Statistical analysis

The sample size was estimated to be able to show differences between the control group and the intervention group of 2 % (alpha error, 0.05; statistical power, 0.80) in mean body fat at the end of the first year. The estimated sample size was 140 children per group; this figure was multiplied by an inflation factor for cluster-randomized trials [51], which was estimated at 1.1264 using measurements from previous studies in body mass index [52]. To examine subgroup differences (e.g., sex, age, ADHD or socio-economic status) under the same conditions, and estimating a 15 % dropout rate, the minimal sample size was estimated at 1600 children (800 per group).

The statistical analysis will be carried out in two phases. In the first, we will verify that the randomization was effective in creating two comparable groups, examining the differences between the control and intervention groups in mean body fat and academic achievement. After that, we will identify outliers and extreme values, and their trustworthiness. Finally, we will check the adjustment of the variables to the normal distribution by using the Kolmogorov–Smirnov test and graphical procedures (normal probability plot).

In the second phase, we will assess the change between the intermediate and final evaluation variables in the first and second years of the study. Mixed regression models [53] will be estimated using each outcome variable as a dependent variable at the end of the first and second years of the study. The different models will be adjusted for baseline values, age and school (cluster), and the interventions will be treated as fixed affects (1 = intervention group, 0 = control group).

The results will be expressed as absolute differences in change in variables between the baseline and the final measurements (confidence interval of 95 %). When the dependent variable is the prevalence of overweight or obesity, the odds ratio will be calculated, together with a 95 % confidence interval.

Because of the different patterns of weight and height, triceps skinfold thickness and body fat, the models will be separately implemented for boys and girls. A separate analysis will also be conducted for ADHD (with or without), socio-economic status, origin (native or foreign), area (urban or rural), and state-funded or private school.

Analyses will be carried out taking into account the CONSORT norms for the publication of the cluster design studies [54], with an intention-to-treat perspective, with children maintained in the group to which they were originally assigned, regardless of the number of sessions they attended.

Results will be considered statistically significant at *P* < 0.05, and the analysis will be performed using the 9.1 version of the SAS statistical package [55]. Mixed generalized lineal models will be used, PROC GENMOD for dichotomous variables, and PROC MIXED for continuous variables.

## Discussion

A panel of experts has recommended that policies be implemented to increase physical activity in children through school-based initiatives. However, resource limitations and public pressure to optimize academic achievement limit opportunities for students to be physically active during the traditional school day. The Movi-Kids programme has several strong points. It uses existing school sports facilities and does not overburden parents and teachers. Neither does it require any changes to the curriculum. Overall, Movi-Kids represents an attractive strategy for increasing physical activity time among schoolchildren, providing activity through structured after-school time.

Several studies have shown that painting the playground in attractive colours and providing permanent or fixed equipment (e.g., nets or climbing adventure parks) increases the time that children are engaged in moderate or vigorous-intensity activities [56, 57]. However, according to a systematic review, higher-quality intervention research is needed to strengthen published findings to inform playtime physical activity interventions [58]. Another innovative aspect of our programme is the inclusion of minor changes in the physical structure of the playground (MOVI-Playground), such as equipment, facilities, paintings, etc., to motivate children to be more active during school break time.

The treatment of ADHD has sometimes been focused mainly on pharmacotherapy (catecholamine release stimulants). Poor tolerance, non-response to treatment and even dependence on these drugs have been extensively reported [59–61]. In addition, the influence of physical exercise on the catecholaminergic systems in children suffering from this disorder has been described [62, 63]. Therefore, it does not seem unreasonable to think that exercise could play an important role as an adjunct to medication, in order to reduce the behavioural problems that interfere with learning and academic progress, and benefit the cognitive performance of children with ADHD [64, 65].

Therefore, in considering data on the prevalence of excess weight in Spanish children, taking into account the evidence of the benefits of physical activity for obesity prevention and also considering the behavioural, neurobiological and genetic links between obesity and ADHD, it seems reasonable that an intervention to promote physical activity based on playground games will be useful for simultaneously controlling ADHD and obesity, thereby improving the academic achievement of students with or without ADHD. Therefore, it is expected that our study will provide information on whether the Movi-Kids programme overcomes some of the potential limitations of physical activity interventions in schoolchildren.

Finally, as this was a cluster-crossover design, the length of the trial will be doubled, and some carryover effect cannot be ruled out; however, it has also been suggested that this design is useful for compensating for the loss of efficiency owing to the clustering.

In summary, this study will evaluate the impact of a multidimensional physical activity intervention (Movi-Kids) in schoolchildren on preventing obesity and improving academic achievement in children from 4 to 7 years with or without ADHD from Castilla-La Mancha, Spain.

## Trial status

The trial is currently recruiting.

## Abbreviation

ADHD: attention deficit hyperactivity disorder
